# Analysis of the mixed teaching of college physical education based on the health big data and blockchain technology

**DOI:** 10.7717/peerj-cs.1206

**Published:** 2023-01-20

**Authors:** Shaoqing Liu, Cun Li

**Affiliations:** Langfang Health Vocational College, Langfang, Hebei, China

**Keywords:** Health big data, Blockchain, Multi-scale cross Attention, Human pose estimation

## Abstract

In the era of health big data, with the continuous development of information technology, students’ physical health management also relies more on various information technologies. Blockchain, as an emerging technology in recent years, has the characteristics of high efficiency and intelligence. College physical education is an important part of college students’ health big data. Unlike cultural classes, physical education with its rich movements and activities, leaves teachers no time to monitor students’ real classroom performance. Therefore, we propose a human pose estimation method based on cross-attention-based Transformer multi-scale representation learning to monitor students’ class concentration. Firstly, the feature maps with different resolution are obtained by deep convolutional network and these feature maps are transformed into multi-scale visual markers. Secondly, we propose a cross-attention module with the multi-scales. The module reduces the redundancy of key point markers and the number of cross fusion operations through multiple interactions between feature markers with different resolutions and the strategy of moving key points for key point markers. Finally, the cross-attention fusion module extracts feature information of different scales from feature tags to form key tags. We can confirm the performance of the cross-attention module and the fusion module by the experimental results conducting on MSCOCO datasets, which can effectively promote the Transformer encoder to learn the association relationship between key points. Compared with the completive TokenPose method, our method can reduce the computational cost by 11.8% without reducing the performance.

## Introduction

The big data of college students’ health is intended to solve various problems encountered in the reality of students’ physical health management, especially the weaknesses in the storage, circulation, utilization, security and other aspects of physical health test data. Through the combination of blockchain technology, college sports hybrid teaching has been realized online, and has become an increasingly important teaching method. It has been accepted by teachers and students because of its convenience and high efficiency. With the great progress of health big data and blockchain, the quality of online physical courses in universities can be guaranteed. In order to better ensure students’ concentration in class and improve the education and teaching quality of online physical courses in universities, a method that can recognize students’ body movements and postures in real time is needed to supervise the quality and quantity of students’ education. Therefore, we conducted research on body posture estimation of students in college physical education courses, and realize real-time supervision by using big data platform and blockchain technology.

In recent years, deep convolutional networks have become an effective tool for learning contextual semantic features, and a large number of excellent convolutional neural network architectures for 2D human pose estimation have emerged (Yuanyuan et al., 2015; Sun et al., 2019; Jing et al., 2022), among which thermographic regression methods (Nie et al., 2019; Wanyi & Deping, 2022; Bin, Ye & Xiaogang, 2022) are the most prominent. Recently, due to the good performance of visual Transformer (Xianjun, 2022) in image recognition, Transformer architecture is introduced into human pose estimation on the basis of convolutional neural network. For example, Trans-Pose (Yang et al., 2021) and TokenPose (Li et al., 2021), establish semantic constraints and connections between key points, and then understand human pose in a global scope, which shows the superior long-distance relationship modeling ability of Transformer. However, these methods try to use a large number of Transformer encoders to establish the association between tokens (Toke-n), which causes learning difficulties to a certain extent (Li et al., 2021). The main reasons can be attributed to two points: Transformer encoders obtain high-resolution fusion feature maps from deep convolutional networks, and some features are up-sampled and fused from low-resolution feature maps (Sun et al., 2019; Wang et al., 2021), resulting in the loss of spatial semantic information. In addition, the Transformer encoder can gradually shift from global to local semantic information, but it needs to stack more encoders, which seriously affects the performance of the model  (Li et al., 2021).

To this end, we propose a cross-attention-based human pose estimation method based on Transformer multi-scale representation learning (CTHPose). The proposed method use the advantages of the low-resolution semantic information, and associate the highest-resolution feature tags output by Transformer encoder with other low-resolution feature maps output by convolutional network in the label space. Firstly, the model uses the deep convolutional network to predict the heat map of key points, and projects it into the label space. At the same time, the feature tags with different resolutions are generated. Then, the proposed cross attention module with multi-scales is used to conduct multiple interactions between the high- and low-resolution feature markers, so that the low-resolution feature markers have stronger identifiability. In order to reduce the redundancy of key point markers and the number of cross-fusion, a mobile key point labeling strategy is adopted. Finally, the fusion module is used again to extract the information of various resolution feature tags by key points. In the first section, we introduce the background and significance of college students’ physical education. In Section 2, we introduce the reference methods and strategies for the methods presented in this article. We will describe our approach in detail in Section 3. Our experimental results and conclusions are presented in Sections 4 and 5 respectively.

Our main contributions are as follows:

1. We propose the cross-attention-based human pose estimation method based on Transformer to monitor students’ class concentration.
2. We model the multiple interactions between the high- and low-resolution features to achieve the better features.
3. We achieve the perfect performance of students posing.

## Related work

The key of the proposed model is to use cross-attention Transformer to model the interaction between multi-scale features, to improve the identifiability of low-resolution global semantic features and to complete the fusion of multi-scale features. Therefore, this section introduces several representative research results from the following aspects:

### Human pose estimation method based on Transformer

Recently, Transformer (Zhao et al., 2022) andits variants (Xianjun, 2022) have been used by researchers for human pose estimation. For example, TransPose  (Yang et al., 2021) uses the attention layer of Transformer to implicitly reveal the dependence relationship between key points, which provides an explanation for the model to infer the global spatial relationship among layers. Inspired by ViT (Xianjun, 2022) (Vision Transformer) model, TokenPose (Li et al., 2021) explicitly models key points as markers to learn the constraint relationship between visual information and key points from images. Both methods require a large number of Transformer encoders, but do not consider low-resolution global semantic features. HRFormer (Yuan et al., 2021) (High Resolution Transformer) uses multi-resolution architecture design and local window self-attention to achieve high-resolution feature representation, which has the characteristics of low memory and low computational cost. This method needs to up-sample the low-resolution features, which can lose the spatial semantic information. The proposed method will further employ the low-resolution global semantic features and make interactive fusion through cross-attention to ensure that the spatial semantic information is not lost.

### Cross-attention

Cross-attention is first used to connect the encoder and decoder in Transformer (Zhao et al., 2022). The cross-attention network (Hou et al., 2019) (CAN) uses cross attention to model the association semantics of class features and query features in the small-sample classification to highlight the target object, which is conducive to subsequent matching. CrossViT  (Chen, Fan & Panda, 2021) uses two different branches to process image tags of different sizes in image classification, and then uses cross attention to fuse class tags. Cross attention has also been used in multi-modal tasks (Lu, Zhou & Ye, 2019; Rodrigues et al., 2022). In this article, we apply the cross-attention for the interaction between different multi-resolution markers to improve the identifiability of low-resolution markers.

## A human pose estimation method based on cross-attention for Transformer multi-scale representation learning

**Figure 1:**
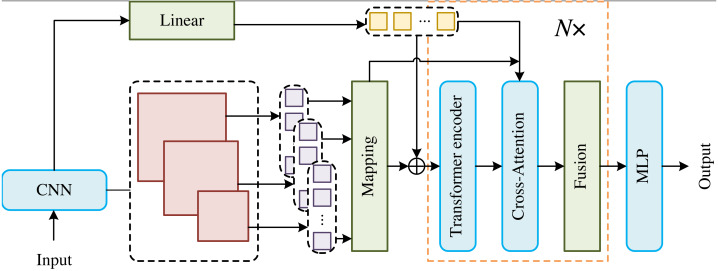
Overall illustration of the proposed CTHPose. In order to effectively utilize the underlying features, we adopt the DCN to build our model. As shown in Fig. 1, the images are first input into the DCN to obtain feature maps with multi-scales, which are uniformly segmented, flattened and linearly projected into visual markers of different resolutions. At the same time, the deep convolutional network and linear function are used to predict the key point markers. Then, the high-resolution visual markers and other low-resolution visual markers are spliced into key point markers and embedded location information, which are used as the main input of the model. Finally, a multi-layer perceptron (MLP) is utilized to reconstruct the keypoint heat map from the two sets of keypoint markers that act on the model output. The main body of CTHPose is divided into three parts: (1) The Transformer encoder is used to establish data flow channels only for the highest resolution visual markers and instead of establishing independent data flow channels for each visual marker of different resolution, so as to form a multichannel parallel network. (2) Inspired by CAN (Hou et al., 2019), other low-resolution visual markers will interact with the highest resolution visual markers in the cross attention with multi-scales to enhance the resolution of low-resolution visual markers. (3) Two groups of key point markers are cross-extracted in the fusion module to obtain visual marker information of different resolutions.

In order to effectively utilize the underlying features, we adopt deep convolution network (DCN) to build our model. As shown in Fig. 1, the images are first input into the DCN to obtain feature maps with multi-scales, which are uniformly segmented, flattened and linearly projected into visual markers of different resolutions. At the same time, the deep convolutional network and linear function are used to predict the key point markers. Then, the high-resolution visual markers and other low-resolution visual markers are spliced into key point markers and embedded location information, which are used as the main input of the model. Finally, a multi-layer perceptron (MLP) is utilized to reconstruct the keypoint heat map from the two sets of keypoint markers that act on the model output.

The main body of CTHPose is divided into three parts: (1) The Transformer encoder is used to establish data flow channels only for the highest resolution visual markers and instead of establishing independent data flow channels for each visual marker of different resolution, so as to form a multichannel parallel network. (2) Inspired by CAN (Hou et al., 2019), other low-resolution visual markers will interact with the highest resolution visual markers in the cross attention with multi-scales to enhance the resolution of low-resolution visual markers. (3) Two groups of key point markers are cross-extracted in the fusion module to obtain visual marker information of different resolutions (Le, 2022; Dai et al., 2022).

### Multi-scale representation tokens in Transformer based on cross attention

**Figure 2:**
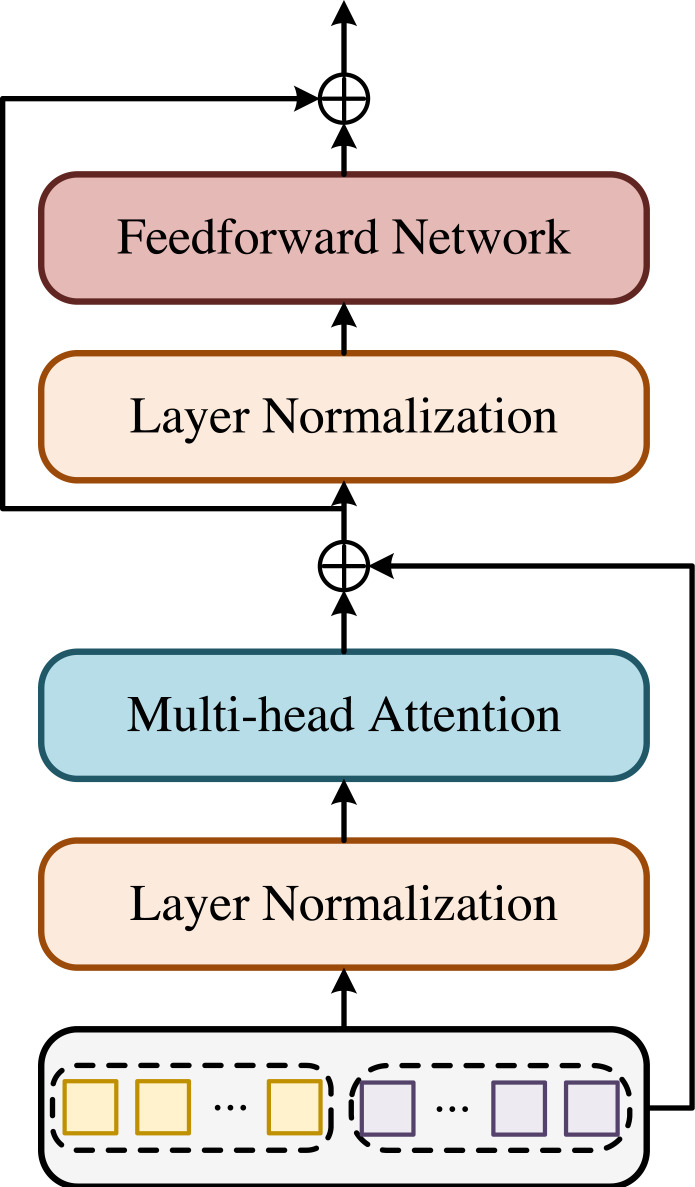
Transformer encoder module. The high-resolution Transformer encoder module is shown in Fig. 2. The input of Transformer encoder (Li et al., 2021) is composed of concatenated highest resolution visual markers and high-resolution keypoint markers. In order to obtain visual markers, the feature map output by the deep convolutional network needs to be transformed into markers. The high-resolution feature map is divided into feature blocks of the same size. Each feature block is then flattened into a 1-dimensional vector, and the embedding vector of -dimension is obtained by linear transformation. In order to obtain fine-grained features, the low-resolution feature map is also divided into smaller blocks of size, which are finally transformed into embedding vectors.

The high-resolution Transformer encoder module is shown in Fig. 2. The input of Transformer encoder (Li et al., 2021) is composed of concatenated highest resolution visual markers and high-resolution keypoint markers. In order to obtain visual markers, the feature map output by the deep convolutional network needs to be transformed into markers. The high-resolution feature map *X*^*l*^ ∈ ℝ^*C*×*H*×*W*^ is divided into feature blocks of the same size *p*_*h*_ × *p*_*w*_. Each feature block is then flattened into a 1-dimensional vector, and the embedding vector ${x}_{v}^{l}$ of *d*-dimension is obtained by linear transformation. In order to obtain fine-grained features, the low-resolution feature map *X*^*s*^ ∈ ℝ^*C*′×*H*′×*W*′^ is also divided into smaller blocks of size ${p}_{h}^{{^{\prime}}}\times {p}_{w}^{{^{\prime}}}$, which are finally transformed into embedding vectors ${x}_{v}^{s}$. The process of feature map transformation into tags is as follows:

**Figure 3:**
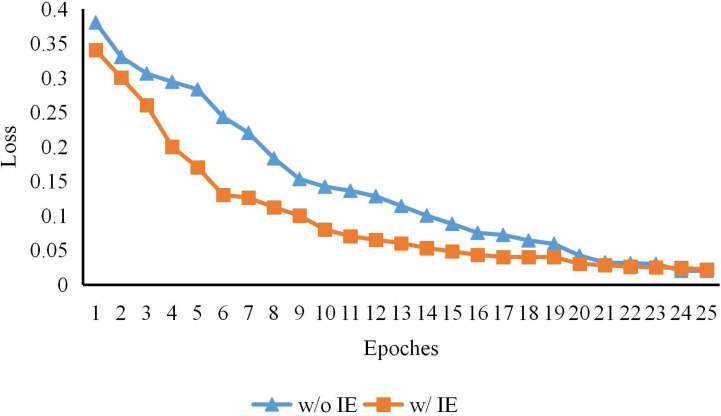
The early training process of CTHPose with and without intermediate estimation. As shown in Fig. 3, with the increase of a certain training period, the convergence speed of the two models in the early training is also very different, but the loss gap is gradually narrowed in the end because the expression ability of the model is limited. This indicates that the key point labeling estimated by the convolutional network provides support for the subsequent Transformer architecture model learning and alleviates the problem of slow Transformer convergence speed.

(1) \begin{eqnarray*}& & {X}^{l}\in {\mathbb{R}}^{C\times H\times W}\rightarrow ^{P}{x}_{v}^{l}\in {\mathbb{R}}^{ \frac{H\times W}{{p}_{h}\times {p}_{w}} \times d}\end{eqnarray*}

(2) \begin{eqnarray*}& & {X}^{s}\in {\mathbb{R}}^{{C}^{{^{\prime}}}\times {H}^{{^{\prime}}}\times {W}^{{^{\prime}}}}\rightarrow ^{P}{x}_{v}^{s}\in {\mathbb{R}}^{ \frac{{H}^{{^{\prime}}}\times {W}^{{^{\prime}}}}{{p}_{h}^{{^{\prime}}}\times {p}_{w}^{{^{\prime}}}} \times d}\end{eqnarray*}

where *H* > *H*′ and *W* > *W*′.*C* and *C*′ respectively represent the channels of the corresponding feature maps. *P* includes segmentation, flattening and linear projection operations. In this article, the keypoint markers that are spliced with high-resolution visual tokens that are called high-resolution keypoint markers and the keypoint markers are not randomly initialized, but the predictive heat map of each keypoint is obtained by deep convolutional network and regressor. Then it is projected into the keypoint marker space.

#### Cross attention with multi-scales.

The proposed multi-scales cross attention module uses the size of squares to represent visual markers from different resolution feature maps, and uses different sizes of circles to represent high resolution and low-resolution key markers. The highest resolution visual markers and high-resolution key markers output by Transformer encoder are recombined with other low-resolution markers to generate new visual markers and key markers, which are used as the input of the cross-attention module. In order to avoid the redundancy of key point markers and multiple fusion of key point markers, the module adopts the moving key point marker strategy.

In order to describe the process of multi-scale cross attention execution, we use *x*^*l*^ and *x*^*s*^ to represent two different resolution tag sequences. The tag contains not only the key tags *x*_*kp*_, but includes visual markers *x*_*v*_, as ${x}^{i}= \left. {x}_{kp}^{i} \right\| {x}_{v}^{i}$, among which *i* = *l*, *s* and || represents concatenation operation. And then we perform the attending operation of crossing between the *x*^*l*^ and *x*^*s*^. We regard the high-resolution tag as the key *k* and value *v* and regard the low-resolution tag as a query *q*, which can be formulated as the following formula:

(3) \begin{eqnarray*}& & q={x}^{l}{W}_{q}\end{eqnarray*}

(4) \begin{eqnarray*}& & k={x}^{s}{W}_{k}\end{eqnarray*}

(5) \begin{eqnarray*}& & v={x}^{s}{W}_{v}\end{eqnarray*}

(6) \begin{eqnarray*}& & A=softmax \left( \frac{q{k}^{T}}{\sqrt{ \frac{d}{h} }} \right) \end{eqnarray*}

(7) \begin{eqnarray*}& & MHA \left( {x}^{l},{x}^{s} \right) =Av\end{eqnarray*}

where ${W}_{q},{W}_{k},{W}_{v}\in {\mathbb{R}}^{d\times \left( d/h \right) }$ are the learnable parameters, *d* is the labeled dimension, *h* is the number of heads and MHA represents the multi-head cross attention function. Since keypoint estimation is a position-sensitive task, the feed-forward network (FFN) is still retained here. Under the condition that layer normalization (LN) and residual connection are introduced, the following formula can be defined based on multi-scale cross attention:

(8) \begin{eqnarray*}& & {y}^{s}=MHA \left( LN \left[ {x}^{l},{x}^{s} \right] \right) +{x}^{s}\end{eqnarray*}

(9) \begin{eqnarray*}& & {z}^{s}=FFN \left( LN \left( {y}^{s} \right) \right) +{y}^{s}.\end{eqnarray*}

In the process of cross attention execution, the markers and key points generated by the highest resolution are never changed and the fusion operation should be further performed. The multi-scales cross attention module is the biggest characteristic which is to use cross attention mechanisms and mobile key strategies in the tag space to realize the interaction between the multi-scale features. Improving the features of low-resolution can be identified and reduce the computational cost of interaction for follow-up attention fusion provide reliable semantic information.

#### Cross-attention fusion.

The highest resolution visual markers are used as keys and values, and the low-resolution keypoint markers are used as queries as input for cross-attention. At the same time, another cross attention fusion module performs the fusion operation of the lowest resolution visual markers and the high resolution key points. The feed-forward network is retained in this module, mainly because there are different and related keypoint markers, while CrossViT (Chen, Fan & Panda, 2021) only needs one class marker in the image recognition task.

### Intermediate estimates of keypoint markers

In this article, deep convolutional networks are used to predict the heat map of key points, which is then projected into the marker space. The detailed calculation steps are as follows: Firstly, the feature map *H* × *W* with the number *C* of channels is transformed into *N* tensor heat maps by 1 ×1 convolution operation. Then, it is transformed into a 1-dimensional vector and mapped to a fixed dimension. CH × WN

The process of predicting key point labeling *z* ∈ ℝ^*N*×*C*^ can be formalized as the following formula:

(10) \begin{eqnarray*}& & y=C \left( x \right) \end{eqnarray*}

(11) \begin{eqnarray*}& & z=MLP \left( F \left( y \right) \right) \end{eqnarray*}

where *x* ∈ ℝ^*C*×*H*×*W*^ represents the feature map output by convolutional neural network, *y* ∈ ℝ^*N*×*H*×*W*^ represents the heat map after 1 × 1 convolutional transformation, *C* represents the convolution operation of 1 × 1, MLP represents the multi-layer perceptron function and *F* represents the dimensionality reduction operation.

## Experiments

### Dataset and implement details

The experimental platform of this article is Ubantu20.04.LTS, programming environment pytorch1.8.1 and Cuda11.1, which are equipped with two Quadro RTX8000 and RTX 3090. All experiments are performed on MS COCO datasets which contains more than 200K images and more than 250K human instances with 17 types of key points. The model is trained only on the COCO train2017 dataset without additional training data and is tested simultaneously on the val2017 dataset and the test-dev2017 dataset, which contained 57K, 150K and 5K samples, respectively.

Object keypoint similarity (OKS) is the evaluation index adopted by keypoint detection task on COCO2017 dataset and the formula is as follows:

(12) \begin{eqnarray*}OKS= \frac{\sum _{i}epx \left( -{d}_{i}^{2}/2{s}^{2}{k}_{i}^{2} \right) \sigma \left( {v}_{i}\gt 0 \right) }{\sum _{i}\sigma \left( {v}_{i}\gt 0 \right) } \end{eqnarray*}

where ${d}_{i}^{2}$ represents the Euclidean distance between the estimated value and the true value of the key point, *s* denotes the target scale, *k*_*i*_ denotes a constant to control the decay of each class of keypoints and a visibility marker *v*_*i*_ represents the true value. Average accuracy (AP) records the average of all values for a given threshold $T\in \left[ 0.5:0.05:0.95 \right] $, for example the calculated AP^75^ represents the performance of *T* = 0.75. When the OKS is greater than this threshold, the position of the key point is detected correctly, otherwise it will be considered to have missed detection and false detection. AP^*M*^ and AP^*L*^ represent the accuracy of model detection of key points in the medium (32^2^ < pixel <96^2^) and large (pixel >96^2^) target areas, respectively.

### Intermediate estimation

On the basis of extracting high-resolution features from the backbone network HRNet-W48-3stage of the model, CTHPose initially estimates the distribution of key points in the label space. As shown in Table 1, although the model parameters have been increased, the scores of AP and AR have been increased to some extent. As shown in Fig. 3, with the increase of a certain training period, the convergence speed of the two models in the early training is also very different, but the Loss gap is gradually narrowed in the end because the expression ability of the model is limited. This indicates that the key point labeling estimated by the convolutional network provides support for the subsequent Transformer architecture model learning and alleviates the problem of slow Transformer convergence speed.

**Table 1:** The intermediate estimation used in CTHPose.

Method	IE	*AP*	*AR*	Parameters
CTHPose	w/	75.5	80.7	23.9
	w/o	75.3	80.5	22.7

**Notes.**

On the basis of extracting high-resolution features from the backbone network HRNet-W48-3stage of the model, CTHPose initially estimates the distribution of key points in the label space. As shown in Table 1, although the model parameters have been increased, the scores of AP and AR have been increased to some extent.

### Results and comparison

Table 2 records the results of this and other state of the art methods. (i) HRNet-w32 (Sun et al., 2019) provides both pre-trained and untrained versions, while the proposed CTHPose is pre-trained in the convolutional network part and untrained in the Transformer part. Compared with the pre-trained HRNet-w32, the average accuracy (AP) and average recall (AR) of CTHPose are increased by 1.4% and 1.1%, respectively, with a slight increase in the computation (GFLOPs) and a slight decrease in the model parameters. Compared with HRNet-w48, CTHPose also achieves better results. Because CTHPose reduces the up-sampling fusion process of low-resolution features, it can interact low-resolution features with high-resolution features in the label space, which improves the low-resolution identifiability. Moreover, the subsequent cross-fusion can eliminate the inaccuracy of up-sampling fusion. (ii) Compared with HRFormer-B (Yuan et al., 2021), CTHPose has a slightly lower score in AP^50^, but it has a slight improvement in overall performance. HRFormer is a Transformer with window self-attention as the main module for extracting deep features of high-resolution network, while CTHPose adopts the cross-attention mechanism to complete the interactive fusion of deep features, which greatly reduces the parameters used and the computational burden. (iii) Compared with TokenPose-L/D6 (Li et al., 2021), CTHPose has the same number of Transformer encoders and achieves better experimental results under the condition of only a small amount of computation (↑6.6%). Compared with TokenPose-L/D24, although some indicators are relatively lower, CTHPose achieves a comparable performance on the entire, while the computational load is reduced by 11.8%. TokenPose-L/D24 uses a 24-layer Transformer encoder, which brings a huge computational burden to the model, while CTHPose uses only six layers. Because the time complexity of the cross-attention mechanism is linear, the inference cost of CTHPose is greatly reduced.

**Table 2:** Comparison with the state-of-the-art models on the COCO validation set.

Method	Pretrain	Parameters	*AP*	*AP* ^50^	*AP* ^75^	*AP* ^*M*^	*AP* ^*L*^	*AR*
HRNet-W32	No	28.5	73.4	89.5	80.7	70.2	80.1	78.9
HRNet-W32	Yes	28.5	74.4	90.5	81.9	70.8	81.0	79.8
HRNet-W48	Yes	63.6	75.1	90.6	82.2	71.5	81.8	80.4
HRFormer-B	Yes	43.2	75.6	90.8	82.8	71.7	82.6	80.8
TokenPose-L/D6	No	20.8	75.4	90.0	81.8	71.8	82.4	80.4
TokenPose-L/D24	No	27.5	75.8	90.3	82.5	72.3	82.7	80.9
CTHPose	No	25.7	75.8	80.2	82.2	72.3	82.7	80.9

**Notes.**

Table 2 records the results of this and other state of the art methods. (i) HRNet-w32 (Sun et al., 2019) provides both pre-trained and untrained versions, while the proposed CTHPose is pre-trained in the convolutional network part and untrained in the Transformer part. Compared with the pre-trained HRNet-w32, the average accuracy (AP) and average recall (AR) of CTHPose are increased by 1.4% and 1.1%, respectively, with a slight increase in the computation (GFLOPs) and a slight decrease in the model parameters. Compared with HRNet-w48, CTHPose also achieves better results. Because CTHPose reduces the up-sampling fusion process of low-resolution features, it can interact low-resolution features with high-resolution features in the label space, which improves the low-resolution identifiability. Moreover, the subsequent cross-fusion can eliminate the inaccuracy of up-sampling fusion. (ii) Compared with HRFormer-B (Yuan et al., 2021), CTHPose has a slightly lower score in, but it has a slight improvement in overall performance. HRFormer is a Transformer with window self-attention as the main module for extracting deep features of high-resolution network, while CTHPose adopts the cross-attention mechanism to complete the interactive fusion of deep features, which greatly reduces the parameters used and the computational burden. (iii) Compared with TokenPose-L/D6 (Li et al., 2021), CTHPose has the same number of Transformer encoders and achieves better experimental results under the condition of only a small amount of computation ( ↑6.6%). Compared with TokenPose-L/D24, although some indicators are relatively lower, CTHPose achieves a comparable performance on the entire, while the computational load is reduced by 11.8%. TokenPose-L/D24 uses a 24-layer Transformer encoder, which brings a huge computational burden to the model, while CTHPose uses only 6 layers. Because the time complexity of the cross-attention mechanism is linear, the inference cost of CTHPose is greatly reduced.

Table 3 records the performance of CTHPose and other methods on the test set. Compared with HRNet, the average accuracy (AP) and average recall (AR) of CTHPose are 1% and 0.7% higher than those of HRNET with fewer parameters and less computational effort, although the score of AP^50^ is slightly different. CTHPose performs better than other methods with Transformer architecture, such as TransPose-H-A6 (Yang et al., 2021) and TokenPose-L/D24 (Li et al., 2021). This is because the proposed CTHPose makes full use of low-resolution global semantic information. Thanks to the linear time complexity of cross-attention, CTHPose can achieve results comparable to TokenPose-L/D24 with less computation. Since small images will be enlarged and fixed to a specific size before input into the model, the image will become extremely blurred, which brings challenges to the model key point location. Compared with TokenPose-L/D24, CTHPose also achieves the same result in AP^*M*^ index. It shows that the performance of CTHPose-s2 is comparable to that of TokenPose-L/D24, the most advanced method for small target areas. According to the CTHPose, we can realize online supervision of college sports teaching, improve the big data platform for college students’ health and improve their physical quality.

**Table 3:** Comparison with the state-of-the-art models on COCO2017 test-dev set.

Method	Parameters	*AP*	*AP* ^50^	*AP* ^75^	*AP* ^*M*^	*AP* ^*L*^	*AR*
HRNet-W48	63.6	74.2	92.4	82.4	70.9	79.7	79.5
TokenPose-H/A6	21.8	75.0	92.2	82.3	71.3	81.1	–
TokenPose-L/D6	9.1	74.9	92.1	82.4	71.5	80.9	80.0
TokenPose-L/D24	11.0	75.1	92.1	82.5	71.7	81.1	80.2
CTHPose	9.7	75.2	92.1	82.5	71.7	81.1	80.2

**Notes.**

Table 3 records the performance of CTHPose and other methods on the test set. Compared with HRNet, the average accuracy (AP) and average recall (AR) of CTHPose are 1% and 0.7% higher than those of HRNET with fewer parameters and less computational effort, although the score of is slightly different. CTHPose performs better than other methods with Transformer architecture, such as TransPose-H-A6 (Yang et al., 2021) and TokenPose-L/D24 (Li et al., 2021). This is because the proposed CTHPose makes full use of low-resolution global semantic information. Thanks to the linear time complexity of cross-attention, CTHPose can achieve results comparable to TokenPose-L/D24 with less computation. Since small images will be enlarged and fixed to a specific size before input into the model, the image will become extremely blurred, which brings challenges to the model key point location. Compared with TokenPose-L/D24, CTHPose also achieves the same result in index. It shows that the performance of CTHPose-s2 is comparable to that of TokenPose-L/D24, the most advanced method for small target areas. According to the CTHPose, we can realize online supervision of college sports teaching, improve the big data platform for college students’ health and improve their physical quality.

## Conclusion

In order to achieve the physical quality of college students and improve the public health level of universities, we rely on health big data and use blockchain technology to continuously improve the mixed teaching level of college sports. Then, we further improved the big data platform for college students’ health to achieve a virtuous circle. In this article, a human pose estimation method based on cross-attention-based Transformer multi-scale representation learning (CTHPose) was proposed. This method uses deep convolutional networks and linear functions to predict the spatial distribution of key point markers and to improve the convergence speed. The main body of the model models the correlation between the highest resolution markers by stacking a small number of Transformer encoders. It then uses the cross attention with multi-scales to modal the interaction between the high-resolution feature maps and the low-resolution feature maps in the marker space, so that the low-resolution feature tags have stronger identification. Thus, the learning efficiency of Transformer encoder is accelerated. At the same time, the excessive redundancy of key point markers was avoided and the fusion times were reduced due to the mobile key point labeling strategy. Experiments on MSOCO show that compared with the other methods, CTHPose only increases a small amount of computation and achieves good results. At present, the lightweight work of human pose estimation model can cost less while testing and improve the inference speed of the model, but basically the premise is to reduce the performance of the model. However, our method still has the defect which can be improved. Our method focuses on the deep learning, while the traditional methods have the great power to solve the issues. In the future, we will explore to combine deep learning and traditional methods to advance the mixed teaching of college physical education.

##  Supplemental Information

10.7717/peerj-cs.1206/supp-1 Supplemental Information 1 Data and code

10.7717/peerj-cs.1206/supp-2 Supplemental Information 2 Computer data

10.7717/peerj-cs.1206/supp-3 Supplemental Information 3 Computer code

## Additional Information and Declarations

### Data Availability

The following information was supplied regarding data availability: The original data and code are available in the Supplemental Files.
